# Supplementary material for: The causal relationship between the human gut microbiota and pyogenic arthritis: a Mendelian randomization study
Source: Front Cell Infect Microbiol. 2024 Nov 26;14:1452480. doi: 10.3389/fcimb.2024.1452480 (PMC11629706; doi:10.3389/fcimb.2024.1452480)
Supplement: Supplementary file 1 [file DataSheet1.zip › mendelian test/ebi-a-GCST90027755.csv_leave_one_out.pdf]

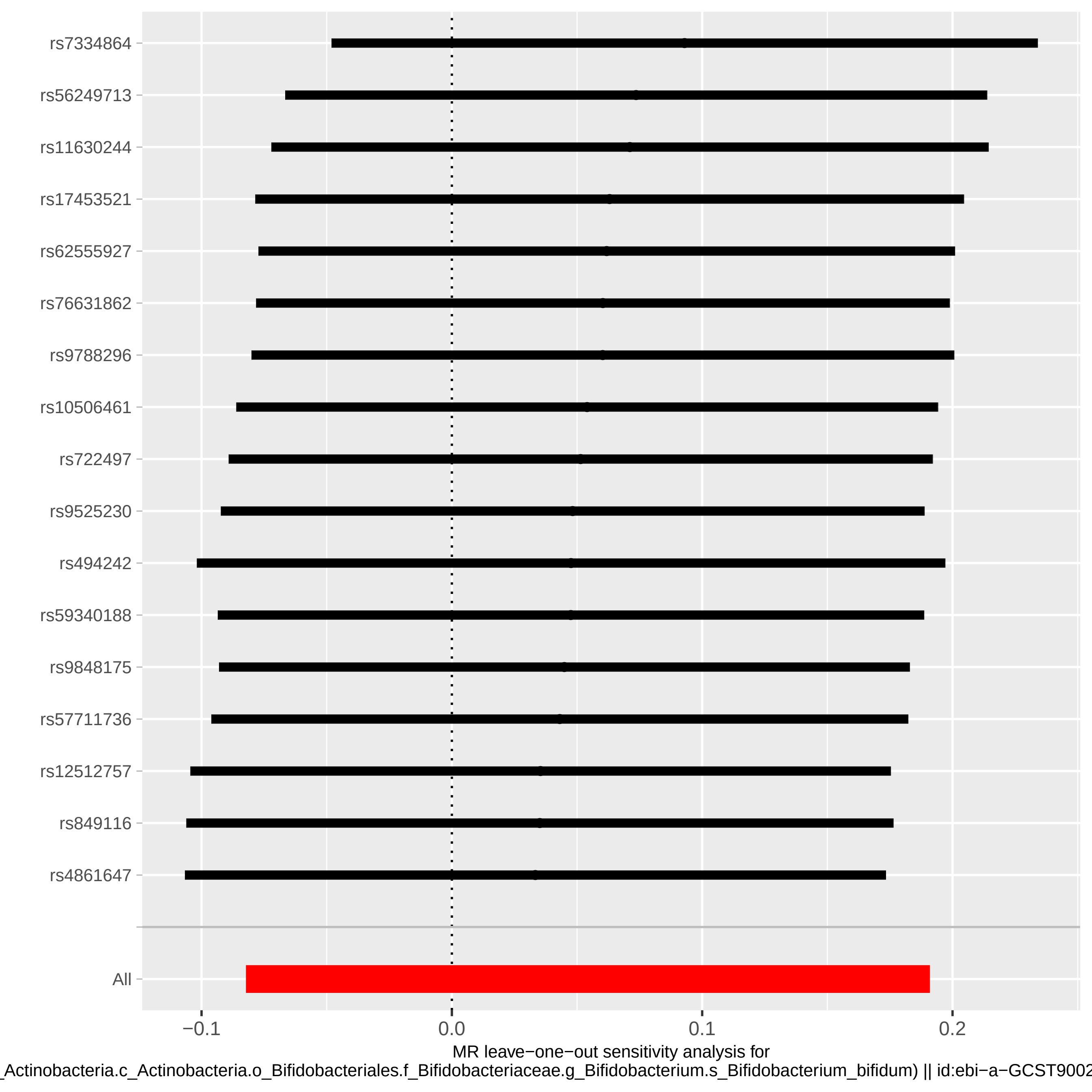
rs7334864
rs56249713
rs11630244
rs17453521
rs62555927
rs76631862
rs9788296
rs10506461
rs722497
rs9525230
rs494242
rs59340188
rs9848175
rs57711736
rs12512757
rs849116
rs4861647
All
−0.1
0.0
0.1
0.2
MR leave−one−out sensitivity analysis for
_Actinobacteria.c_Actinobacteria.o_Bifidobacteriales.f_Bifidobacteriaceae.g_Bifidobacterium.s_Bifidobacterium_bifidum) || id:ebi−a−GCST9002
